# Labor induction with randomized comparison of cervical, oral and intravaginal misoprostol

**DOI:** 10.1186/s12884-021-04196-4

**Published:** 2021-10-27

**Authors:** Masoumeh Dadashaliha, Somayeh Fallah, Monirsadat Mirzadeh

**Affiliations:** 1 Obstetrics and Gynecology, Department of Obstetrics and Gynecology, Children Growth Research Center, Research Institute for Prevention of Non-Communicable Diseases, Qazvin University of Medical Sciences, Qazvin, Iran; 2 Children Growth Research Center, Research Institute for Prevention of Non-Communicable Diseases, School of Nursing and Midwifery, Qazvin University of Medical Sciences, Qazvin, Iran; 3 Community Medicine, Metabolic Disease Research Center, Research Institute for Prevention of Non-Communicable Diseases, Qazvin University of Medical Sciences, Qazvin, Iran

**Keywords:** Misoprostol, Labor induced, Term birth

## Abstract

**Background:**

This study attempts to evaluate the safety and effectiveness of 50μgm intracervical misoprostol in comparison with intravaginal and sublingual for the induction of labor at term pregnant women.

**Methods:**

This study is designed as a parallel clinical trial study. Three hundred and fifteen term pregnancies requiring induction of labor were treated with the maximum used misoprostol intracervical, sublingual, and vaginal doses. Participants were randomly allocated into three groups of 105. The dose was repeated every 4 h until adequate uterine contraction and Bishop Score were achieved. The duration of induction to births, time to the active phase, the rate of births, and the need for caesarean section were compared in three groups. Additionally, labor course and side effects were recorded and analyzed**.** Data were analyzed using SPSS software. A significance level of *p* <  0.05 was considered for statistical analyses.

**Findings:**

Labor was successfully induced in all cases most (63%) of which required a single dose of misoprostol. Ninety-three (93.0%, *p* <  0.05) cervical participants proceeded to vaginal births. This figure was also the same in the vaginal and sublingual group of 83 cases (83.0%). The other 41 cases received caesarean section with more indications of failure to progress and meconium-stained liquor. The results indicated that 278 (92.7%) births were achieved in less than 10 h. Time from start of medication to the active phase of labor and childbirth was 3.01 ± 0.86 and 6.1 ± 1.3 h in the Cervical group, 4.2 ± 0.66 and 8.4 ± 0.92 h in the sublingual group, and 5.06 ± 1.1 and 9.2 ± 1.5 h in the vaginal group respectively (*p* < 0.001). The Caesarean rate was lower in the cervical group than in the two other groups (*p* = 0.05). No significant differences were observed between the study groups in terms of Apgar score and meconium-stained amniotic fluid. Furthermore, no maternal and neonatal complications were observed.

**Conclusion:**

In addition to the sublingual and intravaginal routes of administration, intracervical misoprostol at a single dose of 50μgm appears to be an effective method for induction of labor in women with an unfavorable cervix. Like all medical interventions, a discussion of the risks, benefits, and alternatives to induction of labor with this medication in each woman should be undertaken before treatment.

**Trial registration:**

This clinical study was approved by the Iranian Registry of Clinical Trials with IRCT ID: IRCT20190415043278N1. Registration date was on May 13, 2019 and May 27, 2019 respectively (http://www.irct.ir).

## Background

Induction of labor (IOA) is an essential vital intervention that reduces undesirable effects. Existing regimens using intravenous oxytocin and prostaglandins have been shown to be effective in inducing labor [1]. According to recent research, carrying a pregnancy past 41 weeks is associated with a statistically significant increase in perinatal morbidity and mortality, as well as an increased risk to the mother [2]. Cervical preparation is one of the most substantial factors in the success of labor induction. Attempting induction with an unripe cervix is difficult and rarely successful [3]. Inducing labor with an unripened cervix can result in induction failure [4] or prolonged labor and childbirth with the use of instruments [5]. This will contribute to low levels of satisfaction of delivery, and also to negative psychological and physical effects [6].

While several methods of cervical ripening before induction have been proposed, prostaglandins are the current agents of choice, as it has been shown that the rate of vaginal births increases within 24 h after labor induction and the need for oxytocin decreases [7, 8]. As well as prostaglandins are effective for inducing cervical ripening and stimulating uterine contractions at various doses and routes of administration, orally or vaginally [8, 9]. In terms of cost and storage requirements, misoprostol has been found to be comparable to the currently approved agent dinoprostone [9, 10].

Misoprostol, a prostaglandin E1 analog has gained popularity as an IOL agent in recent years, since it was developed and marketed in the United States in the 1980s, mainly to prevent peptic ulcer disease caused by the use of nonsteroidal anti-inflammatory drugs [8, 11]. The use of misoprostol in obstetrics has sparked a lot of interest since its accidental discovery of triggering uterine contractions in early pregnancy. The FDA revised misoprostol’s original labeling in April 2002 and approved its use in pregnancy [8]. Misoprostol has some potential benefits over other prostaglandins. It is stable at room temperature, cheap, and can be given orally, vaginally, sublingually, and buccally.

To this day, no unique dosage or administration method has been recorded without causing such side effects. However, in Cochrane’s research, the optimal dosage is 25μgm per 4 to 6 h to soften the cervix, which is correlated with the lowest rate of uterine hyperstimulation. One of the unusual, but risky complications associated with the use of this drug is uterine rupture [11]. Various trials have been conducted that compare the types of misoprostol and labor outcomes. The previous studies further found that vaginal misoprostol, compared to cervical dinoprostone and oxytocin, is a more powerful induction alternative. In these trials, the dosage used ranged from 25 μgm every 2 to 3 h to 50 μgm every 4 h to 100 μgm every 6 to 12 h. Besides, it was not able to author that, with or without a change in fetal heart rate, higher doses were associated with uterine tachysystole. On the other hand, misoprostol dosage reduction did not affect the drug’s efficacy decrease [5], and there was no significant difference between the two groups in neonatal admission to the NICU and the neonatal Apgar score [12].

Studies have shown that 50 μgm doses decrease the time interval among contractions and oxytocin requirements and improve vaginal childbirths rate compared to 25 μgm doses; however, the safety of this dose is still uncertain [13].

There are limited data available regarding the safety, effectiveness, and feasibility of administering cervical misoprostol in routine clinical practice. Therefore, we developed a standard cervical misoprostol protocol with the maximum dose (50-ugm) and assessed its safety and effectiveness with vaginal misoprostol and sublingual misoprostol as an induction agent in women who participated in this study.

## Methods

This parallel clinical trial study was conducted at two hospitals in the city of Qazvin, Iran, from July 2019 to September 2020. Three hundred and fifteen participants were selected for this study, of which 105 participants were randomly allocated to each group. Kosar Hospital was a public teaching hospital, while Mehregan Hospital was a private one. The Research Ethics Boards of both hospitals approved this clinical trial and before participating in the study an informed consent form was provided and signed by all volunteer participants. This clinical study was also approved by the Iranian Registry of Clinical Trials with IRCT ID: IRCT20190415043278N1, dated May 27, 2019 (http://www.irct.ir).

Inclusion criteria consisted of singleton pregnancy, gestational age 37 weeks or greater, cephalic presentation, live fetus, cervical Bishop Score ≤ 5, estimated fetal weight < 4000 g, and intact membranes. Women were excluded in case of premature rupture of membranes, placenta previa, placenta abruption, fetal malformations, severe preeclampsia, and abnormal fetal heart rate tracings or signs of active labor at admission and previous uterine scars. Other exclusion criteria were the presence of contraindications for the use of PG analogs, including glaucoma, asthma, epilepsy, and allergy. The misoprostol used in this study (Cytotec, Searle, England) was an analog of prostaglandin E1 in 200 mg tablets. Tablets were divided into quarters for the application of labor induction and each portion containing 50μgm Prostaglandin. Tablets were subdivided through a tablet splitter. The pill cutter used in this study was a Doctor Mode P12 brand which is a plastic splitter commonly available in Iran. The dimensions of the tablet cutter box were 4.0 cm, 4.0 cm, and 7 cm. The metal blade of the cutter had a thickness of 0.30 mm at the middle point. The tablets were placed at the closest point towards the hinge of the cutter inside the designated area on the base plate of the cutter which was parallel to the horizontal plane along the x-axis. There was an axis of symmetry (primary axis) in the direction of the cutting blade with a length of 3.5 cm. The tablets were split along the point-line on a tablet surface. The presence of scores on a tablet surface could increase the chance of obtaining accurate subdivisions, especially if the scores are deep and are present on both faces. In some researches, the accuracies and precisions of the splitting devices have been reported between 94 to 100 and 29.6% respectively [14].

The women were divided into three groups for induction with cervical, sublingual and vaginal misoprostol, respectively. Participants undertake obstetric examinations, including ultrasound and Bishop score at admission upon entering the delivery room and was recorded by one of the midwives. If the Bishop score was less than five before administering preparation, the woman was scheduled for induction of labor, then each participant was given an option by random allocation of software. Misoprostol 50 mcg tablets were administered by a gynecologist to the cervical canal, and the same dose was placed in the posterior fornix or sublingual for induction. To avoid information bias in the estimation of vaginal examination, three midwives were appointed to be responsible for vaginal examination as their vaginal examinations were coordinated with each other before the outset of the study. Prior to each approach, a fetal CTG was conducted for fetal wellness, and dosing was repeated every 4 h until 3 or more uterine contractions lasting 40 s a minute, or when the maximum of 4 doses has been reached.

In the absence of active labor 6 h after the final dosage of misoprostol or if cervical dilation did not advance for 2 h, it was an indication of failure to induce labor so that intravenous oxytocin could be used for augmentation, or if failure to progress, fetal distress, meconium-stained liquor had been diagnosed, caesarean section was performed. Maternal vital signs and gastrointestinal symptoms were evaluated every 2 h. As for safety, continuous fetal and maternal monitoring and progress of labor were recorded on the program.

The primary outcome measures were time from the first administration of misoprostol to labor initiation and induction to childbirth and total doses of misoprostol applied. Secondary outcomes were vaginal births, drug side effects, and fetal/neonatal complication rates. Adverse effects included disorders of uterine contractility (tachysystole and hyperstimulation syndrome), gastrointestinal symptoms such as nausea, vomiting, hyperthermia, diarrhea, and headache. We adopted the definition of Heuser et al. [15] in which uterine tachysystole was defined by any occurrence of five or more contractions within 10 min, averaged over three consecutive 10-min periods, uterine hyperstimulation syndrome was defined as uterine tachysytole with concurrent fetal heart rate decelerations or bradycardia, hypertonus as a single contraction with the duration of at least 2 min. Neonatal outcome consisted of the rates of 5-min Apgar score < 7, umbilical artery/venous pH, presence of meconium and NICU hospitalization. In cases where the Apgar score was less than 7, arterial blood gas cord blood data were collected.

A demographic questionnaire containing information such as age, parity, abortion history, and body mass index (BMI) was initially filled out. The second questionnaire was a partogram, in which labor progress was recorded during labor and after the births. Several data, such as dilatation and effacement of the cervix, fetal head station, Bishop score estimation, status of water bags, side effects of medications, birth weight, Apgar score, and amniotic fluid transparency were collected.

According to the same study by Marsdal et al. [16], considering power = 80%, α = 5%, and also 10% attrition risk in sample size, 105 women in each group were selected. The participants were randomly assigned into three study groups. Random allocation was carried out using the simple randomization method, and assignment sequences were documented on the provided form before the commencement of the research as either cervical, sublingual, and vaginal. Randomization was performed using random allocation software. Although blinding of intervention might not have been possible, blinding of outcomes measurement and/or statistical analysis was ensured. For this purpose, the type of intervention was written in accordance with the assignment sequence and was enclosed within the opaque envelopes. The second investigator prepared the sealed envelopes to ensure the allocation concealment. Tablets were administered by a registered gynecologist and a labor report was recorded by 3 registered midwives who conducted vaginal examinations and coordinated with each other prior to the outset of the study.

Statistical analysis was processed using SPSS software (version 20), and *P* < 0.05 was considered significant. The continuous, and categorical data were described as the mean ± standard deviation (SD), and the frequency and percentage respectively. Dichotomous variables were compared between the groups using the Chi-square test or Pearson, and continuous variables were analyzed using the one-way ANOVA test. The differences in the induction-onset of labor intervals were evaluated by Tukey Test**.** Covariance analysis (ANCOVA) was used to compare the effects of some variables on time to active phase. Cohen’s effect size was similarly used to evaluate the efficiency of our interventions for reducing time to active phase and delivery. The effect size was interpreted according to Cohen’s definition (Cohen 1988). The ranges of the effect sizes were as follows: ≤0. 2 (minimal to small), 0. 2–0. 5 (small to moderate), 0. 5–0. 8 (moderate to large), and ≥ 0. 8 (large).

## Results

**Fig. 1:**
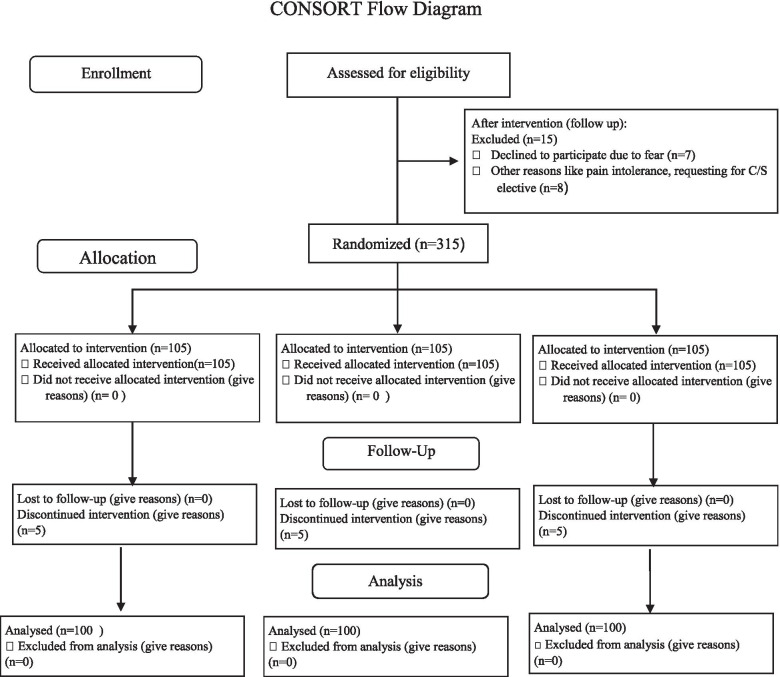
CONSORT Flow Diagram

In the first study, 315 healthy women were chosen to participate in the study, of which 15 women after the commencement of the study, 5 people in each group, in total 15 people changed their mind not to take apart due to their different concerns. Then 300 women were allocated for cervical preparation into three intervention groups. Figure 1 is summarized of the flow map. Comparisons of the participants’ features of the three groups are demonstrated in Table 1. The three groups had no substantial differences before the interventions in maternal height, weight, gestational age, parity, maternal age, abortion history, and Bishop score. In addition, most of the participants in these three groups (cervical-30.3%, sublingual-34%, vaginal 34.9%) were primigravida. In the cervical group, the mean gestational age at admission was 39.4 ± 0.78 weeks, and in the other two groups, 39.1 ± 1.9. Post-dates were the most common indication for induction in three classes. In the sample group, most women had a Bishop score of 1–2, representing 0.79% (cervical) and 0.9% (sublingual, vaginal), respectively, and this was not statistically significant (*p* = 0.38). The baseline characteristics were comparable in three groups (*p* > 0.05). For most of the items, the differences between the three groups were minimal.

**Table 1:** Baseline demographic data and clinical characteristics

Characteristic variable	Cervical group	Sublingual grou	Vaginal group	Total
Age(y) (M ± SD)	29.3 ± 5.	28.5 ± 4.7	28.4 ± 5.1	28.7 ± 5.08
Parity				
Primiparity	53(30.3%)	61(34.9%)	61(34.9%)	175(100%)
Multipara	47(37.6%)	39(31.2%)	39(31.2%)	125(100%)
Gravidity				
Primigravida	48(31.45%)	50(32.7%	55(35.9%)	153(100%)
Multigravida	52(35.4%)	50(34%)	45(30.6%)	147(100%)
Previous abortion: n (%)				
Yes	21(42.9%)	11(22.4%)	17(34.7%)	49(100%)
No	79(31.5%)	89(35.5%)	83(33.1%)	251(100%)
Gestational Age (M ± SD)	39.4 ± 0.78	39.1 ± 1.9	39.1 ± 1.8	39.2 ± 1.6
BMI (kg/m2) (M ± SD)	23.6 ± 3.3	24.4 ± 3.4	24.03 ± 3.7	24 ± 3.5
Bishop Score (M ± SD)	0.79 ± 0.49	0.90 ± 0.83	0.92 ± 0.76	0.87 ± 0.71
Birth weight (g) (M ± SD)	2.2 ± 0.52	2.2 ± 0.62	2.2 ± 0.56	2.2 ± 0.57

*BMI* Body mass index

The effects of three interventions on time between administration to the active phase and time between preparations to childbirth can be seen in Table 2. There were significant differences between groups in time to births and time to active phase. Two-group comparisons using the Tukey Test revealed that the meantime to the active phase (*p* < 0.001) and time to births (*p* < 0.001) in the cervical group were significantly lower than in the vaginal and sublingual groups. Moreover, in the sublingual community, the meantime to the active process (*p* < 0.001) and time to births (*p* < 0.001) were significantly lower than in the vaginal group. One hundred and eighty-nine (63%) women needed only one dose of misoprostol. Two doses were needed for the other hundred and eleven (37%). The statistics also indicated that the caesarean frequency was different in the three groups (*p* = 0.05). Failure to progress (4 cases) and meconium-stained liquor (3 cases) were the reasons for caesarean section in the cervical group. In the sublingual community, 17 cases of caesarean section were identified with indications of non-progress (five cases) and meconium-stained liquor, respectively (12 cases). Finally, in the vaginal group, 11 and 6 cases were accompanied with meconium-stained and failure to progress, respectively. No case with fetal distress or uterine hypertonicity was observed.

**Table 2:** Comparisons of labor and delivery outcomes in three groups

Variable	Cervical group	Sublingual group	Vaginal group	*p*-value
Time to Active phase (hours) (M ± SD)	3.01 ± 0.86	4.2 ± 0.66	5.06 ± 1.1	< 0.001
Time to delivery (hours) (M ± SD)	6.1 ± 1.3	8.4 ± 0.92	9.2 ± 1.5	< 0.001
Single dose of misoprostol: n (%)	96(96%)	63(63%)	28(28%)	0.000
Mode of delivery: n (%)				
Vaginal delivery	93(93)	83(83)	83(83)	0.05
Caesarean	7(7.0)	17(17.0)	17(17.0)	
Gastrointestinal implication	0	0	0	–
Hyperstimulation	0	0	0	–
Tachysystol	0	0	0	–
Failure to progress: n (%)				
Yes	4(4%)	5(5%)	6(6%)	0.81
No	94(94%)	95(95%)	96(96%)	

According to the effect size time to active phase (1.6, 1.8 and 0.9, respectively), the difference between the three interventions is in the large area. Based on the size of the effect for the time until births, the difference between cervical, sublingual and cervical with vaginal is in the large area and the difference between sublingual and vaginal is in the small to the moderate area. (Table 3(.

**Table 3:** Effect size and mean difference among the three intervention groups

	Cervical group vs sublingual group	Cervical group vs vaginal group	Sublingual group vs vaginal group
Time to Active phase (hours) (Mean difference ± SD)	1.2 ± 0.13	2.05 ± 0.12	0.8 ± 0.11
Effect size	1.6	1.9	0.8
Time to delivery (hours) (Mean difference ± SD)	1.74 ± 0.18	2.5 ± 0.16	0.76 ± 0.12
Effect size	1.5	2.2	0.64

The comparison of Apgar infants in the three groups is displayed in Table 4. In the three classes, the first-minute Apgar score did not vary (*p* = 0.1) but it was different after 5 min (*p* < 0.001). The Apgar score of one and 5 min between the three groups was not significant. In the NICU, no infants were hospitalized. Moreover, fetal distress and death in the infant were not observed.

**Table 4:** Effects of interventions on Apgar score and meconium -stained Liquor

Variable	Cervical group	Sublingual group	Vaginal group	*p*-value
First minute Apgar (M ± SD)	9.0 ± 0.00	8.6 ± 1.7	8.7 ± 1.2	0.11
Five minute Apgar (M ± SD	10.00	10.00	10.00	0.00
Birth weight > 3500 g: n (%)				
YES	26 (26%)	27(27%)	26(26%)	0.98
NO	74 (74%)	73(73%)	74(74%)	
Meconium-stained Liquor: n (%)				
Yes	3(11.5%)	12(46.2%	11(42.3%)	0.46
No	(35.4%)	88(32.1%)	89(32.5%)	
Fetus Distress	0	0	0	–
Need to NICU	0	0	0	–
Death	0	0	0	–

In this analysis, covariance analysis (ANCOVA) was used and according to this table to try to account for the effects of age, Bishop score, and groups on time to the active phase. The period to the active phase is substantially altered by Bishop Scores and intervention groups, but age had no significant impact. Adjusted R Squared = .461 suggests that the Bishop variable and the intervening variable can estimate approximately 46% of the time for active phase changes (Table 5).

**Table 5:** Effects of variable on time to active phase

Variable	Mean Square	F	Adjusted *p*-value
Bishop Score	7.669	8.991	.003
Age	.841	.986	.321
Group	106.036	124.315	.000

## Discussion

This study compares cervical misoprostol with intravaginal and sublingual misoprostol in homogeneous groups. This study aims to compare the safety and effectiveness of intracervical, vaginal, and sublingual regimens. We discovered shorter mean intervals between the start of induction and childbirth in group A, as well as a higher proclivity for vaginal births without significantly raising the maternal and fetal complications and adverse effects, which is consistent with other previous studies on the topic [17].

Previous studies have shown that sublingual intake or intravaginal misoprostol administration is successful for labor induction. However, the ideal dosage and route of administration, remain contentious. Here the intracervical route of administration is recorded and a favorable outcome in labor induction is achieved. In this study, the majority of 189 (63%) of our participants required a single induction dose of 50μgm, which is similar to other investigations [17, 18]. In the cervical groups, the time from initial administration to regular contractions was 3.01 ± 0.86 h, a figure better than the intravaginal and sublingual results of the same dose of misoprostol [19, 20]. Approximately 92.7% of women achieved vaginal births below 10 h and 7.3% achieved vaginal induction up to 10 h, of which the cervical community reported the least time to childbirth with a mean of 6.1 ± 1.3 h. In the cervical group, the rate of spontaneous vaginal births (93%) appeared to be better than that reported by Souizi et al. (64.5%) [21], Veena (76.8%) [20], Girija (60%) [22], Jahromi [23] using routes of administration, sublingual or vaginal. On the other hand, there was no note of the time difference between the vaginal and sublingual groups in the study variation. However, the time intervals associated with the sublingual community were shorter for just 1 h (*p* < 0.001). It is also possible to display intracervical and sublingual administration as a more effective route than vaginal agents. Gattás’s study_ A randomized, placebo-controlled trial of 12.5μgm sublingual and 25μgm vaginal dose administration found that in the sublingual community, the duration between the first dose of misoprostol and the onset of labor was shorter [24]. Sublingual misoprostol was also found in Ayati’s study to be as successful as vaginal misoprostol for term labor induction. However, sublingual misoprostol has the benefit of easy administration and may be more fitting than vaginal misoprostol [25]. Consequently, the reports were in accordance with previous findings [26]. Although the findings were not in harmony with Feitosa’s report, which found that 25ugm sublingual misoprostol administration was neither more effective nor safer than the same vaginally administered dose [27]. Due to the small sample size, it may be possible to use a higher Bishop score of samples (≤ 6), with low doses of misoprostol that do not allow conclusive conclusions to be drawn. The required intracervical dose of misoprostol is almost 2 times lower than the intravaginal or sublingual dosage [18, 19, 27]. While there is a lack of pharmacokinetic evidence on the local administration of misoprostol [28]. It is conceivable that intracervical misoprostol directly enters the target organ, thus optimizing the local impact and decreasing systemic absorption [18]. In addition, it was found that the sublingual route of administration has an area similar to vaginal administration under the curve, but more rapid absorption and higher peak levels than either vaginal or oral administration, which may support the findings of our literature [29].

The conclusion of the several studies [18, 19, 22] indicated that participants who were treated with misoprostol were suffering from gastrointestinal experiences, tachysystole, and hyperstimulation which was the result of misoprostol dosage. The rates were 0.0% with 25μgm dosage [30], 18–25% with 50μgm [17]. However, in this study, we did not have such experiences because the uterine contractions were monitored continuously, and misoprostol was used if there was no contraction or less than three contractions in 10 min. In all three groups, the evaluation of the caesarean indication was similar, including failure to progress and meconium-stained liquor. Like studies by Souizi et al. (7%), Dasgupta and Roudsari et al. (10%), the caesarean rate was 7% [21, 31, 32]. No statistically significant differences have however been reported. Fortunately, the neonatal result was positive in all three groups as all neonates were born alive with a median Apgar score of 9, 10 at 1 and 5 min respectively and no child was hospitalized in NICU.

## Conclusion

Our findings indicate that intracervical administration of misoprostol is effective in inducing labor without side effects on women during pregnancy and any obvious adverse effects on the fetus. In females with the unripened cervical disorder, it may decrease labor period and time to childbirth. The effects of sublingual routes of administration on women and the fetus were similar or more beneficial. Further studies are needed on the use of higher-dose of cervical misoprostol on primiparas to provide a better direction for ongoing research on this subject.

## Data Availability

The dataset used and/or analyzed during this study are available from the corresponding author on reasonable request.

## Abbreviations

FDA: US Food and Drug Administration
BMI: Body Mass Index.
